# Seroprevalence of influenza A H1N1 and influenza D viruses in ruminants in Qatar

**DOI:** 10.1016/j.onehlt.2025.101005

**Published:** 2025-02-26

**Authors:** Hadeel T. Zedan, Tasnim Alziftawi, Abrar Abdalla, Hadi M. Yassine

**Affiliations:** a Biomedical Research Center, QU Health, Qatar University, PO Box 2713, Doha, Qatar; b College of Health Sciences, QU Health, Qatar University, PO Box 2713, Doha, Qatar

**Keywords:** IDV, IAV, H1N1, Seroprevalence, Ruminants, HAI

## Abstract

**Background:**

Influenza is among the most common viruses affecting humans and many animals worldwide. While influenza A (IAV) and D (IDV) viruses are associated with respiratory disease in humans and animals, respectively, their prevalence in the Middle East is unknown.

**Methods:**

Here, we assessed the seroprevalence of IDV and IAV/H1N1 in 331 ruminants (including camels, sheep, cattle, and goats) in Qatar. Sera samples were collected from ruminants in different farms and titrated by hemagglutination inhibition (HAI) assay.

**Results:**

We showed a high prevalence of IDV in all ruminants, ranging between 55 and 84 %, with the highest rates seen in sheep and cattle. The rates were much lower for IAV, ranging between 6 and 8 %, but were not detectable in goats. HAI titers of IDV-seropositive samples ranged between 20 and 2560, whereas IAV titers ranged between 20 and 640.

**Conclusions:**

Our study provides the first serological evidence of IDV and IAV/H1N1 in ruminants in Qatar. These results underscore the need for further investigation into the role of ruminants in influenza virus transmission.

## 1 Introduction

Influenza is one of the most common respiratory viruses in the world; it belongs to the *Orthomyxoviridae* family, which are enveloped negative-sense single-stranded RNA viruses with a segmented genome [1]. Four types of influenza viruses have been identified: A, B, C, and D, which are distinguished based on the infected host, genetic variation, and epidemiology [2]. Influenza A virus (IAV) and influenza B virus (IBV) are the most common types affecting humans and are responsible for causing seasonal epidemics in the winter [3]. IAV and IBV genomes encode for various proteins, including the viral glycoproteins haemagglutinin (HA) and neuraminidase (NA), which facilitate viral entry and release, respectively. Further, IAVs are characterized by their ability to infect a wide range of hosts, with aquatic birds as the primary reservoir. IAVs can also infect various mammalian species, including ruminants, swine, horses, poultry, and humans, contributing to their broad host range and zoonotic potential. Contrarily, IBV is restricted to humans and often associated with mild symptoms [4]. Influenza C virus (ICV) is less common and less severe than IAV and IBV. It affects humans, mainly causing lower respiratory disease with milder cold-like symptoms [4]. In 2011, the influenza D virus (IDV) was discovered in swine which was shown to share approximately 50 % sequence similarity with the ICV [5]. It primarily infects cattle, with no cases reported in humans [6]. It also has been found in other ruminants, such as sheep, goats, and swine, posing a potential risk to livestock health and raising concerns about its zoonotic potential [5].

Although swine and birds are the main reservoirs for H1N1, it can also infect other species of ruminants like cattle, which have been reported in some seroepidemiology studies [7]. Such findings highlight the importance of surveillance across multiple species to fully understand the ecology and transmission dynamics of H1N1. Several studies assessed the seroprevalence of IDV in ruminants in various countries. However, there is a paucity of data regarding the influenza virus subtypes circulating in ruminants in our region, especially IAV/H1N1. To date, no such studies have been conducted in Qatar or the Middle Eastern and North African (MENA) countries. Therefore, the present study aimed to determine the seroprevalence of IDV and IAV in different ruminant species in Qatar farms.

## 2 Materials and methods

### 2.1 Sample collection and ethical compliance

331 sera samples were collected from ruminants, including camels, cows, sheep, and goats. All the animals were healthy. Sera samples were collected over one year (December 2019–October 2020) from different farms in Qatar. Sera samples were stored at −80 °C before usage. This study has obtained Qatar University approval IBC No. QU-IBC-2020/073 before sample collection. This research project posed no risk to animals, and their rights and welfare were not harmed. Therefore, all consent requirements were waived from the QU committee before sample collection.

### 2.2 Stock virus generation

Influenza viruses, including IDV (strain D/bovine/France/5920/2014) and IAV/H1N1 (strain A/California/7/2009), were obtained through BEI Resources, NIAID, and NIH. IDV was propagated on human rectal tumor cells HRT-18G (ATCC CRL-3609) in Dulbecco's modified Eagle's medium (DMEM; Gibco™, USA) supplemented with 1 μg/ml TPCK (tosylsulfonyl phenylalanyl chloromethyl ketone)-trypsin (ThermoFisher, MA, USA) at 37 °C, 5 %CO_2_ for 5 days. IAV was propagated in Madin-Darby canine kidney (MDCK, ATCC CCL-34) in modified Eagle's medium (MEM; Gibco™, USA) supplemented with 1 μg/ml TPCK-trypsin at 37 °C, 5 %CO_2_ for 5 days. Viruses were stored at −80 °C until further used. The virus titers were determined by the 50 % tissue culture infective dose (TCID50) method, as previously described [8]. Viral RNA was extracted from cell supernatants using QIAamp Viral RNA Mini Kit (Qiagen, Germany) following the manufacturer's protocol. RT-qPCR was used to determine the viral RNA using Luna Universal Probe qPCR kit (New England Biolabs, MA, USA). For IDV, probe sequence FAM-cactacatttcccagctgttgactcc-BHQ1, forward primer cctgaaaagattgcgaatgcag, and reverse primer gttgggtttcagtgccattc were used. For IAV/ H1N1, probe 56-FAM/cagccagcaatrttrcatttacc/3|abkfq, forward primer agaaaagaatgtaacagtaacacactctgt and reverse primer sequence tgtttccacaatgtargaccat were used.

### 2.3 Hemagglutination inhibition (HAI) assay

Briefly, sera were pretreated with receptor-destroying enzyme (RDE; Denka Seiken, Japan) following the manufacturer's instructions and then adsorbed with a 20 % suspension of chicken red blood cells (RBCs) for 1 h at 4 °C. HAI assay was performed following the guidelines of the World Organization for Animal Health manual [9]. Each virus used in the HI assay was standardized to contain 8 HA units/50 μl. The treated serum (1:10) was heat-inactivated at 56 °C for 30 min and then serially diluted two-fold with PBS. Then, 50 μl of diluted serum (50 μl) were mixed with 50 μl of standardized IDV or IAV and then incubated for 30 min at room temperature. Finally, 50 μl of 1 % chicken RBCs were added and incubated for 30 min. HI titres were determined as the reciprocal of the highest dilution of the serum inhibiting agglutination.

### 2.4 Statistical analysis

Statistical analyses were carried out using Graph Pad Prism (version 10.3). χ2 test and one-way analysis of variance (ANOVA) were used to compare IAV and IDV seroprevalences between the different ruminants. A *p*-value ≤.05 was considered significant.

## 3 Results

**Fig. 1:**
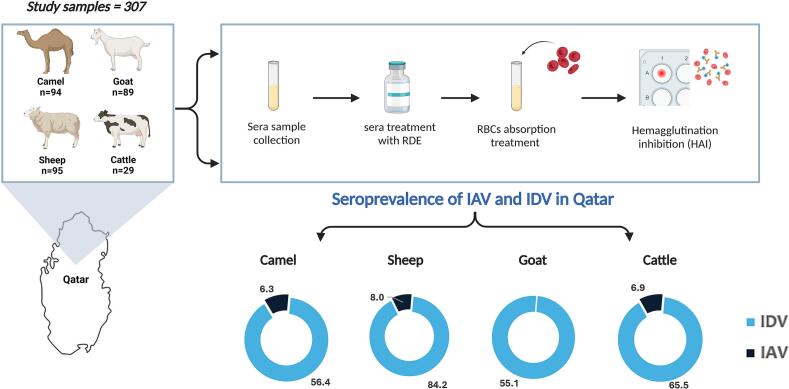
Graphical representation of the study design for assessing the seroprevalence of influenza D virus (IDV) and influenza A virus (IAV) in different ruminants in Qatar. The study included 307 samples collected from camels (*n* = 94), sheep (*n* = 95), goats (*n* = 89), and cattle (*n* = 29). Sera samples were collected from ruminants, treated with receptor-destroying enzyme (RDE), and absorbed with chicken red blood cells (RBCs) to eliminate non-specific inhibitors. Hemagglutination inhibition (HAI) was performed to determine IDV and IAV titers. The figure was created using BioRender. (For interpretation of the references to colour in this figure legend, the reader is referred to the web version of this article.)

A total of 307 sera samples collected from ruminant animals, including camels (*n* = 94), sheep (*n* = 95), goats (*n* = 89), and cattle (*n* = 29), were tested for IDV and IAV antibodies. Among the tested samples, 65.5 % (201/307) were seropositive for IDV, whereas 5.0 % (11/219) were seropositive for IAV/H1N1. The highest seropositivity rate for IDV was observed in sheep (84.2 %), followed by cattle (65.5 %), camels (56.4 %), and goats (55.1 %). HAI titers observed in IDV-seropositive samples ranged between 20 and 2560, with the highest titers seen in camels and sheep sera (Table 1). HAI titers in IAV-seropositive samples ranged between 20 and 640, with sheep showing the highest titers. IAV antibodies were detected in camels (5/80; 6.3 %), sheep (4/50: 8.0 %), and cattle (2/29; 6.9 %), but not in goats. (See Fig. 1.)

**Table 1:** Seropositivity rates of influenza D virus (IDV) and influenza A virus (IAV)/ H1N1 in camels, sheep, goats, and cattle in Qatar.

IDV							
Animal Species	Number of tested samples	Number of positive samples	Seroprevalence (%)	HI titer range	OR	95 % (CI)	*p*-value
Camels	94	53	56.38	80–2560	RF		<0.001
Cattle	29	19	65.52	20–160	1.47	0.62–3.50	0.38
Goats	89	49	55.06	40–1280	0.95	0.53–1.70	0.86
Sheep	95	80	84.21	40–2560	4.13	2.08–8.20	<0.001
Total	307	201	65.6	20–2560			


IAV							
Animal Species	Number of tested samples	Number of positive samples	Seroprevalence (%)	HI titer range	OR	95 % (CI)	*p*-value
Sheep	50	4	8.00	20–640	RF		0.67
Camels	80	5	6.25	20–40	0.77	0.19–3.00	0.70
Cattle	29	2	6.90	20–80	1.84	0.42–7.99	0.42
Goats	60	0	–	–	0.00	–	0.99
Total	219	11	5.0	20–640

In addition, camels were more likely to be seropositive than goats (OR = 0.95, *p* = .89). In contrast, cattle, and sheep had 1.5 and 4.1 higher odds, respectively (OR = 1.47, *p* = .38 for cattle and OR = 4.13, *p* < .001 for sheep) of being seropositive to IDV compared with camels (Table 1). Further, sheep had a higher risk of being infected by IAV than camels and goats (OR = 0.77 for camels and OR = 0.0 for goats). However, cattle had a 1.8 higher risk of infection with IAV than sheep.

**Fig. 2:**
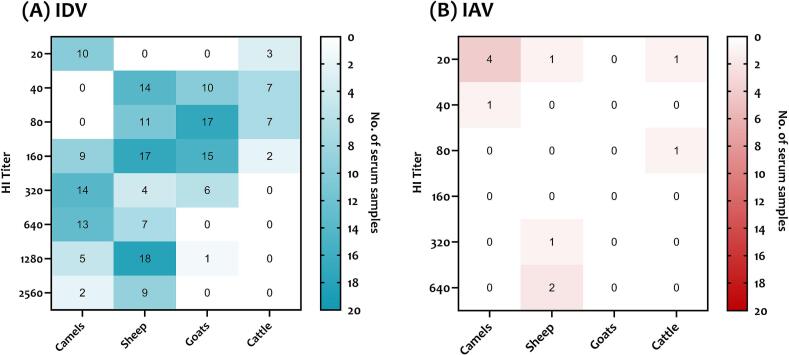
Heatmap analysis of seropositive ruminants for influenza D virus, IDV (A) and influenza A virus, IAV (B). According to the legend with a blue or red gradient located to the right of each figure, the number of seropositive animals is indicated by dark blue or red for a high number of seropositive animals and by light blue or red for a low number of samples. The HI titer titer is represented on the y-axis. (For interpretation of the references to colour in this figure legend, the reader is referred to the web version of this article.)

Heatmap analysis of seropositive samples showed that most seropositive sheep and goats had IDV HAI titers between 40 and 160 (Fig. 2A). The highest IDV HAI titers were detected in sheep (between 1280 and 2560). High titers were also detected in seropositive camels ranging between 160 and 2560, whereas the lowest titers were seen in cattle. As for IAV, only sheep showed high HAI titers ranging between 320 and 640 (Fig. 2B). Other IAV seropositive ruminants, including camels and cattle, had low HAI titers ranging between 20 and 80.

## 4 Discussion

In this study, we determined the seroprevalence of anti-IDV and anti-IAV antibodies in domestic ruminants in Qatar. Our results showed that IDV and IAV/ H1N1 circulate in ruminants, including camels, sheep, and cattle. Goats were seropositive for IDV but not for IAV/ H1N1. We found a higher IDV seropositivity rate in all ruminants, ranging between 55 and 84 % compared with other studies. Seroprevalence studies across various regions have demonstrated a wide range of IDV exposure among ruminants, depending on species, region, and study conditions. The seroprevalence of IDV is generally highest in cattle herds, often exceeding 50 % in surveyed populations. In our study, IDV seroprevalence in cattle was 65.5 %. A study in Ireland reported a seroprevalence of 54.7 % in cattle [10]. Similarly, studies in France, Italy, and Luxembourg reported varying seroprevalence rates, with cattle showing higher exposure (∼80.2 %) than small ruminants [11,12]. High IDV seroprevalence rates were also reported in North America [13]. Studies from countries like China identified significant seropositivity in cattle (91.4 %), although data from other ruminants remains limited [14]. In Africa, the seroprevalence ranges between 3.9 % and 20.9 %, reflecting regional variations in virus transmission dynamics and livestock practices [15]. However, rates in other ruminants like sheep and goats are generally lower, often below 5 % in most surveyed regions  [16]. Here, we found higher seroprevalence rates in these ruminants, especially sheep. The environmental conditions in the MENA region, including high temperatures and arid landscapes, may influence the stability and transmission of IDV, potentially affecting seroprevalence rates. Also, livestock movement across borders for trade and religious festivities, such as the Hajj pilgrimage, may facilitate the spread of IDV, necessitating coordinated regional surveillance efforts.

IDV exhibits antigenic diversity among its lineages, which may influence seroprevalence estimates by affecting cross-reactivity in serological assays. Antigenically distinct IDV strains have been identified in various regions worldwide [11] and previous studies have shown that differences among IDV lineages can impact immune recognition, potentially leading to variability in measured antibody responses [17]. As a result, the seroprevalence observed in this study may be influenced by the specific IDV strain used for serological testing, with possible underestimation or overestimation of antibody prevalence depending on the antigenic similarity between circulating strains and the test antigen. This highlights the importance of considering lineage-specific variations when interpreting serological data. In this study, we utilized the D/bovine/France/5920/2014 strain for the HI test, which was isolated in France and belongs to the D/swine/Oklahoma-like lineage and has been shown to share substantial antigenic similarity with more recent IDV isolates. Therefore, this strain remains relevant for assessing exposure to IDV during 2019–2020. Still, the presence of antigenically diverse IDV strains in the region cannot be excluded, which may have affected the sensitivity of our assay.

To date, most studies focused on assessing the seroprevalence of IAV/H1N1 in pigs and other animals closely associated with humans since ruminants are not the primary reservoirs [7]. Studies on backyard pigs have reported varying IAV/H1N1 seroprevalence, with notable seropositive cases in Africa and Asia [18]. Further, one study from Ohio has studied the seroprevalence of H1N1, H3N2, and H3N8 in dogs, reporting a low prevalence of those viruses in dogs [19]. Globally, there have been few documented cases of H1N1 in cattle and other ruminants, with seroprevalence typically associated with isolated outbreaks or cases of interspecies transmission. For example, seroconversion in cattle species in Ireland was observed in outbreaks of respiratory disease, diarrhea, and milk drop syndrome [20]. In that study, they assessed 84 paired acute and convalescent cattle serum samples and reported a 56.5 % seroconversion rate in convalescent sera, indicating that cattle are naturally susceptible to human IAV. Here, IAV/H1N1 seroprevalence ranged between 5 % and 8 %. No similar studies were documented in other regions, which highlights a significant gap in understanding the role of ruminants as potential hosts or reservoirs for H1N1. In addition, our study used the 2009 H1N1 strain, which represents the pandemic H1N1 (pH1N1) lineage that has been the dominant circulating strain in both human and animal populations since 2009. While using a more recent strain could refine the seroprevalence estimates, the chosen strain is still likely to capture a significant portion of past exposure, particularly in the context of conserved antigenic sites.

In conclusion, both IDV and IAV/H1N1 were found to be circulating in different ruminants in Qatar. The zoonotic potential of both viruses remains uncertain; however, the prevalence observed in this study highlights the need for further investigation, particularly among individuals with close contact or occupational exposure to these animals. A key limitation of this study is the lack of detailed metadata on individual animal histories, exposure sources, and movement patterns, which restricts our ability to perform a more in-depth analysis of factors influencing seroprevalence across different locations. Therefore, more research is required to clarify the involvement of these viruses in bovine respiratory disease and to assess any possible risks to human health, particularly considering the ongoing H5N1 avian influenza outbreaks, which are posing significant global concern, affecting wild birds, poultry, and mammals, with sporadic cases in humans.

## Funding

This work was funded by 10.13039/501100004252Qatar University, BRC Internal Funds 2020, and Qatar National Research Fund (QNRF), grant MME03-1128-210032.

## CRediT authorship contribution statement

**Hadeel T. Zedan:** Writing – review & editing, Writing – original draft, Validation, Software, Methodology, Investigation, Formal analysis, Data curation. **Tasnim Alziftawi:** Writing – original draft, Methodology, Investigation. **Abrar Abdalla:** Writing – original draft, Methodology, Investigation. **Hadi M. Yassine:** Supervision, Resources, Project administration, Funding acquisition, Conceptualization.

## Declaration of competing interest

The authors declare that they have no known competing financial interests or personal relationships that could have appeared to influence the work reported in this paper.

## Data Availability

Data will be made available on request.
